# Editorial: Biological Basis and Therapeutics of Behavioral and Psychological Symptoms of Dementia

**DOI:** 10.3389/fpsyt.2022.838962

**Published:** 2022-02-10

**Authors:** Deana Davalos, Antonio Teixeira, Manabu Ikeda

**Affiliations:** ^1^Department of Psychology, College of Natural Sciences, Colorado State University, Fort Collins, CO, United States; ^2^Columbine Health Systems Center for Healthy Aging, Colorado State University, Fort Collins, CO, United States; ^3^Department of Psychiatry & Behavioral Sciences, University of Texas Health Science Center, Houston, TX, United States; ^4^Faculdade Santa Casa BH, Belo Horizonte, Brazil; ^5^Department of Psychiatry, Graduate School of Medicine, Osaka University, Suita, Japan

**Keywords:** dementia, BPSD, behavioral symptoms, psychological symptoms, biomarkers, neurocognitive decline

Behavioral and psychological symptoms of dementia (BPSD), also referred to as neuropsychiatric symptoms, include a wide range of symptoms affecting perception, thought content, mood, and behavior (1). Understanding these symptoms is critical as BPSD often present early in neurocognitive disorders, are some of the most persistent symptoms, and represent the leading cause for one's loss of independence for dementia patients (2). The eight articles that examine BPSD in this Research Topic aim to dissect the biological basis of BPSD, explore life span behaviors that may improve BPSD outcomes and inform the reader of how we may better serve those with BPSD and their caregivers with more thoughtful understanding of their underlying factors.

As is the trend with most clinical disorders, there is increased interest in determining the earliest indicators of pathology to help expedite diagnosis in the prodromal stage and use of potentially disease-modifying therapies. For frontotemporal dementia (FTD), Alzheimer's disease (AD) and related dementias (ADRD), BPSD are some of the first indicators of dementia to emerge. Research is evolving that suggests that there are biomarkers and patterns of cognition that may inform on risk for neurocognitive decline and even measures that may help distinguish types of neurocognitive decline early in the neurodegenerative process. Pai et al. explore one type of perception, temporal perception (TP), that has been associated with difficulty managing independent activities of daily living. Temporal processing deficits have been implicated in dementia in past research, but the current study investigates the use of TP as a means to differentiate two types of prodromal dementia, prodromal AD and prodromal dementia with Lewy Bodies (DLB). Satake et al. explore the consequences, i.e. psychosis, of different types of perceptual disturbances. Hallucinations and delusions are well-documented in dementias. However, discriminating late-onset psychosis from ADRD-associated psychosis has been particularly difficult. The authors found that biomarkers, such as amyloid positron emission tomography and cerebrospinal fluid, may shed light on cases in which very late-onset schizophrenia-like psychosis may in fact represent prodromal AD. Li et al. continue the exploration of key biomarkers associated with the development of dementia. Specifically, their findings suggest that there is a relationship between polymorphisms in specific genes (including BIN1 and APOE) and AD, with notable sex differences in terms of each gene and susceptibility to AD. This collection of studies target biomarkers and symptoms that may help not only detect early symptoms of dementia but also specific types of dementia that may require different therapeutic strategies.

While identification of risk factors is critical in the diagnosis of ADRD, understanding strategies that are associated with prevention or delaying their course are equally important. For instance, healthy lifestyle behaviors are associated with a substantially lower risk for AD, with adherence to several specified healthy behaviors found to reduce the risk of developing AD by 60% (3). Conversely, psychosocial stressors and mood disorders have also been associated with increased risk of ADRD (4). In the current Research Topic, Lam et al. explore the effects of mindfulness-based interventions to address symptoms of depression and anxiety in older adults. Findings suggest that dispositional mindfulness is associated with a lower level of mood symptoms in older adults in Hong Kong and that high mindful awareness may reduce the adverse effects of chronic physical morbidity on mental health in both healthy older adults and those with cognitive impairment. In the work presented by Liu and Wu, the authors tackle a very different type of behavior – bilingualism – that may be associated with not only building cognitive reserve but also modifying the pathway of neurocognitive decline. The authors review the brain and biochemical mechanisms of bilingualism that may delay the onset of dementia and the perspective that bilingualism can be considered as a prescribed intervention with no side effects.

In the final set of articles, researchers seek to inform us on the relevant issue of how BPSD affects those with ADRD, caregivers, institutions and society. Macfarlane et al. discuss an issue that has received widespread attention in recent years, the potential overprescribing of psychotropic medications in the treatment of BPSD in residential settings. The authors note, in Australia, up to 91% of people living with ADRD live their final years in supported residential facilities (5). While medications (psychotropic, acetylcholinesterase (AChE) inhibitors (AChEi), mood stabilizers and antidepressants) are the most common intervention, the authors found that psychosocial person-centered care interventions delivered by multidisciplinary dementia-specific behavior support programs are clinically effective, resulting in improved BPSD and related caregiver distress. The interaction of BPSD and caregiver burden is explored further in research focused specifically on DLB. DLB is thought to represent the second most common type of neurodegenerative dementia and is associated with high levels of BPSD and accompanying high levels of caregiver burden (6). While it is clear that caregiver burden is high, the factors in DLB that affect feelings of burden remain relatively unknown. Kanemoto et al. sought to classify BPSD to discover which factors are linked to caregiver burden. The authors uncovered three BPSD factors similar to AD (psychosis, affection, hyperactivity), a DLB-specific factor (wakefulness) and each BPSD factor contributing to caregiver burden in a different way. In the final article, Burley et al. also explore the idea of reconceptualizing and redefining BPSD to better inform care based on the perspectives of families and caregivers. The authors propose that a reconceptualization of BPSD terminology is needed to understand and de-stigmatize BPSD symptoms and improve the quality of care and support for caregivers.

Across studies, a general theme emerges suggesting that greater understanding of specific factors of BPSD is needed to help researchers, clinicians, institutions and caregivers address the development and treatment of these symptoms. This collection provides current views of how we may be able to use technology to better identify risk factors, non-pharmacological interventions to potentially stave off the development of BPSD, and improved terminology and classification to better treat symptoms and assist those who care for individuals with BPSD.

## Author Contributions

All authors listed have made a substantial, direct, and intellectual contribution to the work and approved it for publication.

## Conflict of Interest

The authors declare that the research was conducted in the absence of any commercial or financial relationships that could be construed as a potential conflict of interest.

## Publisher's Note

All claims expressed in this article are solely those of the authors and do not necessarily represent those of their affiliated organizations, or those of the publisher, the editors and the reviewers. Any product that may be evaluated in this article, or claim that may be made by its manufacturer, is not guaranteed or endorsed by the publisher.
